# Mitochondrial F-ATP Synthase Co-Migrating Proteins and Ca^2+^-Dependent Formation of Large Channels

**DOI:** 10.3390/cells12192414

**Published:** 2023-10-07

**Authors:** Anna B. Nikiforova, Yulia L. Baburina, Marina P. Borisova, Alexey K. Surin, Ekaterina S. Kharechkina, Olga V. Krestinina, Maria Y. Suvorina, Svetlana A. Kruglova, Alexey G. Kruglov

**Affiliations:** 1 Institute of Theoretical and Experimental Biophysics, Russian Academy of Sciences, Institutskaya 3, 142290 Pushchino, Russia; nikiforanna@yandex.ru (A.B.N.); byul@rambler.ru (Y.L.B.); aakmpb@mail.ru (M.P.B.); katya.kypri@gmail.com (E.S.K.); ovkres@mail.ru (O.V.K.); 2 Branch of the Shemyakin—Ovchinnikov Institute of Bioorganic Chemistry, Russian Academy of Sciences, Prospekt Nauki 6, 142290 Pushchino, Russia; alan@vega.protres.ru; 3 State Research Centre for Applied Microbiology and Biotechnology, 142279 Obolensk, Russia; 4 Institute of Protein Research, Russian Academy of Sciences, Institutskaya 4, 142290 Pushchino, Russia; marrruko@yandex.ru; 5 Institute of Basic Biological Problems, Russian Academy of Sciences, Institutskaya 2, 142290 Pushchino, Russia; krugsa@rambler.ru

**Keywords:** mitochondrial complex V, F-ATP synthase, monomer, dimer, permeability transition pore, F-ATP synthase, prohibitin, methylmalonate–semialdehyde dehydrogenase, high-conductance channel

## Abstract

Monomers, dimers, and individual F_O_F_1_-ATP synthase subunits are, presumably, involved in the formation of the mitochondrial permeability transition pore (PTP), whose molecular structure, however, is still unknown. We hypothesized that, during the Ca^2+^-dependent assembly of a PTP complex, the F-ATP synthase (subunits) recruits mitochondrial proteins that do not interact or weakly interact with the F-ATP synthase under normal conditions. Therefore, we examined whether the PTP opening in mitochondria before the separation of supercomplexes via BN-PAGE will increase the channel stability and channel-forming capacity of isolated F-ATP synthase dimers and monomers in planar lipid membranes. Additionally, we studied the specific activity and the protein composition of F-ATP synthase dimers and monomers from rat liver and heart mitochondria before and after PTP opening. Against our expectations, preliminary PTP opening dramatically suppressed the high-conductance channel activity of F-ATP synthase dimers and monomers and decreased their specific “in-gel” activity. The decline in the channel-forming activity correlated with the reduced levels of as few as two proteins in the bands: methylmalonate–semialdehyde dehydrogenase and prohibitin 2. These results indicate that proteins co-migrating with the F-ATP synthase may be important players in PTP formation and stabilization.

## 1. Introduction

Despite fifty years of extensive research, the molecular structure of the PTP is still a matter of debate [1,2,3,4,5,6,7,8,9,10,11,12,13]. PTP models proposed earlier, such as heterooligomeric complexes of adenine nucleotide translocase, voltage-dependent anion channel (VDAC), peripheral benzodiazepine receptor, cyclophilin D (CyPD), creatine kinase, and Bax/Bcl-2 proteins [14,15,16]; or phosphate transporter and CyPD [17]; or VDAC, CyPD, and spastic paraplegia 7 [18], were rejected based on the data on the genetic ablation of putative PTP components [19,20,21,22,23,24,25,26].

Recently, novel PTP models have been proposed, which comprise an oligomer of the F-ATP synthase subunit c [5,11] and a dimer/oligomer of the F-ATP synthase as a core component [2,4,27]. The first model implies the Ca^2+^-dependent separation of the matrix-directed subcomplex F_1_ from the membrane-embedded subcomplex F_O_ comprising the ring of subunits c, which forms a large pore. According to the second model, a PTP is formed as a result of conformational changes in the F-ATP synthase dimer (or oligomer), which are induced by the binding of Ca^2+^ to the subunit β within the F1 subcomplex and propagate to the inner membrane through the peripheral stalk, particularly, by the oligomycin sensitivity-conferring protein (OSCP) [2,4,27]. Novel findings indicate that the interaction of ATP synthase inhibitory protein IF1 with OSCP protects cancer cells from PTP-dependent apoptosis [28]. Moreover, the subunits g and f of the mammalian F-ATP synthase and g and e of the yeast F-ATP synthase are essential for stability and high-conductance activity in planar lipid membranes [8,9,29,30,31]. Additionally, the decline in the level of subunit f in mammalian mitochondria reduces the size of PTP [32].

However, thermodynamically, the pore formed by the oligomer of the F-ATP synthase subunit c should be extremely unstable [3]. Additionally, it was reported that the PTP persisted in mitochondria lacking the subunit c [33], though with reduced channel conductance [7].

The F-ATP synthase di-(oligo-)mer model was also challenged by the data, indicating that the stabilization of the F-ATP synthase dimer inhibits PTP opening [11] and that the deletion of genes encoding the subunits b, OSCP [12], e, and g [8] has a minimal effect on PTP formation. In addition, the F-ATP synthase monomer was shown to be sufficient for the creation of megachannel-like conductance in membranes [10], while human mitochondria devoid of an assembled F-ATP synthase were able to undergo the PTP opening [8]. Nevertheless, the latter conclusion was challenged by the latest data, according to which the destruction of the ATP synthase (via the deletion of subunits c and δ) or removal of adenine nucleotide translocase in mitochondria led to the activation of the CsA-sensitive proton channel rather than PTP, indicating a different molecular structure of these entities [34].

Thus, the molecular structure of PTP is still to be established. Failures in attempts to solve this problem may be due to the following reasons. The number of F-ATP synthase complexes (including monomers, dimers, and oligomers) in a mitochondrion is about 15,000, while the number of PTP complexes is one or two and does not exceed nine [35]. Hence, PTP opening is a rather rare event, which may require the presence of minor proteins in the inner membrane, the intermembrane space, or the matrix. Indeed, besides OXPHOS complexes, the inner membrane contains multiple proteins immersed in and anchored to the lipids or associated with the matrix and the intermembrane surfaces of the membrane. One can assume that some of these proteins associate with a dimer or a monomer of the F-ATP synthase complex in the presence of Ca^2+^ and participate in the formation and stabilization of the PTP complex.

In fact, the PTP in mitochondria does not close spontaneously unless Ca^2+^ is removed and/or strong PTP antagonists are added, indicating a high stability of the PTP structure [36,37,38]. In addition, mitochondrial megachannels (MMC) in mitoplasts are characterized by stable states of conductance and clear transitions between them [39,40,41], while isolated and purified dimers and monomers of the F-ATP synthase in artificial lipid membranes demonstrate quickly changing conductance with unclear states [2,4,10].

We hypothesized that PTP opening in mitochondria before the separation of (super)complexes and other proteins via blue-native gel electrophoresis could increase the level of partner proteins associated with F-ATP synthase dimers and monomers after separation and, thus, increase the stability and activity of channels formed upon the incorporation of dimers/monomers into lipid membranes.

Here, we compared the data of mass spectrometric analysis and measurement of the channel activity of the F-ATP synthase (dimers and monomers) isolated from control mitochondria and mitochondria in which the PTP was opened. The results confirm the role of F-ATP synthase-associated proteins in PTP formation.

## 2. Materials and Methods

Isolation of rat liver and heart mitochondria. All manipulations with animals were performed in accordance with the Helsinki Declaration of 1975 (revised in 1983), national requirements for the care and use of laboratory animals, and protocol 9/2020 of 17 February 2020 approved by the Commission on Biological Safety and Bioethics at the ITEB RAS. Rat liver mitochondria (RLM) were isolated using a standard differential centrifugation procedure [42]. The homogenization medium contained 220 mM mannitol, 70 mM sucrose, 10 mM HEPES (pH adjusted to 7.4 with Trizma Base), 1 mM EGTA, and 0.05% BSA. The mitochondrial pellet was washed three times with a medium devoid of EGTA and BSA. Final pellets were resuspended in this medium to yield ~70 mg protein/mL. Rat heart mitochondria (RHM) were isolated in the same buffer in a similar way, except that the mitochondrial pellet was washed twice. The concentration of isolated RHM was approximately 20 mg protein/mL. The mitochondrial protein was assayed via the Biuret method using BSA as a standard.

Mitochondria were incubated at 37 °C either in a KCl-based medium (KCl-BM) (125 mM KCl, 20 mM sucrose, 10 mM HEPES (pH adjusted to 7.3 with Trizma Base), 2 mM KH_2_PO_4_, 2 mM MgCl_2_, and 10 µM EGTA) or in a sucrose–mannitol-based medium (SM-BM) (220 mM mannitol, 70 mM sucrose, 10 mM HEPES (pH 7.3), 2 mM KH_2_PO_4_, 2 mM MgCl_2_, and 10 µM EGTA) supplemented with 5 mM malate plus 5 mM pyruvate. The quality of isolated mitochondria, indicated by the respiratory control coefficient, was assessed using Oroboros Oxygraph-2k (Innsbruck, Austria) by measuring the oxygen consumption rates before, in the course, and after the termination of phosphorylation of known amounts of ADP. In the studies, the respiratory control coefficient was not less than 5.

Registration of PTP opening. The opening of PTP in isolated mitochondria was registered as Ca^2+^-dependent, EGTA- and CsA-sensitive high-amplitude swelling. Mitochondrial swelling was determined by measuring a decrease in A_550_ in mitochondrial suspension using a plate reader (Infinite 200 Tecan, Tecan Global Headquarters, Männedorf Switzerland) and 96-well plates. Both intact and swollen mitochondria were extracted from the suspension by 3 min centrifugation at 15,000× *g* (0–4 °C).

Blue native electrophoresis (BN-PAGE). BN-PAGE was performed as described [40]. The solubilizing buffer (0.75 M e-Amino-n-caproic acid, 50 mM Bis-Tris/HCl (pH 7.0), and 10% digitonin (Sigma, St. Louis, MO, USA)) was added to mitochondria sedimented from control and PTP samples, and the suspension was kept on ice for 20 min. After 10 min centrifugation at 10,000× *g*, the supernatant was supplemented with 5% Serva Blue G dissolved in 1 M e-Amino-n-caproic acid (Sigma, St. Louis, MO, USA). Samples were applied onto 3–13% gradient gel, 70 µg of the sample per lane. Electrophoresis was performed on ice at 0–4 °C. An HMW Calibration Kit for Native Electrophoresis (Sigma–Aldrich, St. Louis, MO, USA) was used as a marker of molecular mass. Two gels were run simultaneously, one for in-gel activity determination and the other for SDS-PAGE and immunoblotting.

Measurement of the in-gel activity of electron transport chain complexes and the F-ATP synthase. The in-gel activity of complexes I, IV, and F-ATP synthase (CI, CIV, and CV) was determined as described [43]. To assess CI activity, gels were stained for ~10–30 min with a buffer containing 100 mM Tris-HCl (pH 7.4), 0.14 mM NADH, and nitroblue tetrazolium chloride (1 mg/mL). To determine CIV activity, gels were stained for 1 h with a buffer containing 10 mM KH_2_PO_4_ (pH 7.4), 3,3′-diaminobenzidine (1 mg/mL), and cytochrome c (0.2 mg/mL). The measurement of ATPase activity included the staining of gels for 16 h with a buffer containing 270 mM glycine, 35 mM Tris (HCL) (pH 7.0), 14 mM MgSO_4_, 10 mM ATP, and 0.2% Pb(NO_3_)_2_. After the incubation of gels with appropriate substrates, the reactions were stopped with 10% acetic acid; gels were washed with water and scanned. CIII activity was defined in accordance with [44].

SDS-PAGE and immunoblotting. The bands of separated complexes were cut out from BN-PAGE gels and applied onto 12.5% SDS-PAGE slabs, followed by electrophoresis and immunoblotting. The molecular mass of proteins was calibrated using Precision Plus Pre-stained Standards markers from Bio-Rad Laboratories (Hercules, CA, USA).

The subunits of ETC complexes were detected using a Total Oxphos Rodent WB Antibody Cocktail (Abcam, Cambridge, UK). The cocktail contained the antibodies against the F-ATP synthase subunit alpha (CV-ATP5A-55 kDa), cytochrome b-c1 complex subunit 2 (CIII-UQCRC2-48 kDa), mitochondrially encoded cytochrome c oxidase subunit I (CIV-MTCO1-40 kDa), succinate dehydrogenase (ubiquinone) iron–sulfur subunit b (CII-SDHB-30 kDa), and NADH dehydrogenase (ubiquinone) 1 beta subcomplex subunit 8 (CI-NDUFB8-20 kDa). Additionally, the F-ATP synthase subunit c was detected using the ATP5G antibody from Abcam (Cambridge, UK). Immunoreactivity was studied using appropriate secondary antibodies conjugated with horseradish peroxidase (Jackson Immuno Research, West Grove, PA, USA). The blots were stained with ECL (Bio-Rad, Hercules, CA, USA) and inspected using the ChemiDoc Touch Imaging System (Bio-Rad, Hercules, CA, USA).

Tandem mass spectrometry (MS-MS) analysis. For each variant of mitochondria and incubation medium, the bands of the F-ATP synthase dimer and monomer, complex I and mitochondrial supercomplexes CI-CIII_2_-CIV_n_ + CI-CIII_2_-CIV_n_ and CIII_2_-CIV_n_ were collected from four BN-PAGE gels of two independent mitochondrial isolations. Each gel contained the same number of control and PTP samples. Protein bands were excised and independently treated with trypsin, chymotrypsin, and proteinase K (Sigma) at 37 °C in a Thermo Mixer thermo shaker (Eppendorf, Hamburg, Germany). The molar enzyme-to-protein ratio was 1/50. The reaction was stopped by the addition of trifluoroacetic acid to the solution. Prior to mass spectrometric analysis, the peptides were separated via reversed-phase HPLC using an Easy-nLC 1000 Nanoflow chromatograph (Thermo Fisher Scientific, Waltham, MA, USA). The separation was carried out in an adsorbent with a particle size of 3.6 μm and a pore size of 300 Å. The column was packed under laboratory conditions at a pressure of 350 atm. The peptides were eluted in a gradient of acetonitrile from 5% to 45% for 180 min; the mobile phase flow rate was 0.27 μL/min.

The mass spectra of samples were obtained using an OrbiTrap Elite mass spectrometer (Thermo Fisher Scientific, Waltham, MA, USA). The method of ionization of peptides was nanoelectrospray. The temperature of the input capillary was 220 °C, and the voltage between the emitter and the input capillary was 1.9 kV. The panoramic mass spectra were shot with a resolution of 60,000 (for *m*/*z* 400). The spectra were recorded in the range of 300–1600 *m*/*z*. Ion fragmentation was carried out via the collision-activated dissociation with an inert gas in a high-energy cell (HCD). The resolution of the device when scanning the fragmentation spectra was 15,000.

MS data analysis and protein quantification. The results of the MS-MS analysis were processed using the commercial programs Thermo Xcalibur Qual Browser and PEAKS Studio 7.5/Xpro based on the provided sequences of rat proteins (Uniprot database, Rattus Norvegicus (by 1 December 2022). The masses of the peptides were determined with an accuracy of at least 5 ppm. The masses of fragments were determined with an accuracy of 0.1 Da. (Residue modifications: fixed modifications—none, variable modifications—oxidation, formylation, and carbamidomethylation.) False discovery rates at peptide and protein levels were 4.9 and 8.4%, respectively. For the analysis of PTP-associated changes in the protein composition of the bands, we selected all true proteins if they were identified at least by one unique peptide with at least 5% coverage of protein sequence or by two unique peptides with at least 1% coverage (Datasets_S01–S07). The abundance of a protein in the band was determined as a relative intensity-based absolute quantification (rIBAQ): the sum of peak intensities of all unique peptides of a protein (IBAQ) divided by the sum of peak intensities of all unique peptides of all true proteins in the band (Dataset_S07).

Electrophysiology. F-ATP synthase dimers and monomers were eluted from excised protein bands of BN-PAGE gels exactly as described by [4]. The elution buffer contained 25 mM tricine, 10 mM MgSO_4_, and 7.5 mM Bis-Tris (pH 7.0), with the addition of 2.5 mM ATP-Tris (pH 7.4) and 1% *w*/*v* n-heptyl β-D-thioglucopyranoside. After overnight rotation at 4 °C, the eluate was centrifuged at 20,000× *g* for 20 min at 4 °C, and the supernatants were collected for reconstitution in electrophysiological analyses. The elution buffer for other mitochondrial complexes and supercomplexes contained 2.5 mM NADH instead of ATP. The electrophysiological properties of mitochondrial complexes and supercomplexes were studied after their insertion into artificial planar lipid membranes. Bilayer lipid membranes were formed according to Muller–Rudin [39] using a solution containing 20 g/L of soybean lecithin (L-α-phosphatidylcholine Type IV–S, Sigma, St. Louis, MO, USA). The surrounding solution contained 0.2 M KCl and 5 mM HEPES-Tris (Sigma), pH 7.3. Voltage clamp conditions were used throughout the experiments. The *cis* compartment was connected to the virtual ground through a Keithle 301 amplifier in the current-to-voltage configuration. The membrane potential was maintained through Ag/AgCl electrodes in 3 M KCl and 2% agar bridges. The data were digitized using a DT2801A board (Data Translation, North Lawrence, OH, USA) via AD–DA converters connected to a PC, which collected the data using the software developed in-house by A.Ya. Silberstein. The agent under study was introduced into the chamber part with the measuring electrode at the same side (*trans*) of the membrane. Ca^2+^ was added on the side of mitochondrial complexes. The sign of the potential on the figures refers to the *cis* side of the membrane. All measurements were performed at room temperature (~22 °C).

Experimental Design and Statistics. In order to increase the accuracy of determination of PTP-related changes in specific activity, channel-forming activity, and protein composition of mitochondrial supercomplexes, to cover a wider range of supercomplexes, and minimize the number of experimental animals, the experimental protocol and approach to data analysis were as follows.

Experimental protocol. Related samples. For each incubation media and mitochondrial type, a common mitochondrial sample was divided into control and PTP parts, supplemented with either EGTA or Ca^2+^, respectively. Two independent mitochondrial samples from four BN-PAGE gels were collected for each pair of samples for MS-MS analysis. Internal controls. The abundance of a protein in the BN_PAGE band was determined as a relative intensity-based absolute quantification (rIBAQ): the sum of peak intensities of all unique peptides of a protein (IBAQ) divided by the sum of peak intensities of all unique peptides of all true proteins in the band. High threshold. In our hands, the inaccuracy of the determination of a well-recognizable protein between technical replicates lies in the range of 10–20% of rIBAQ. We applied a much higher threshold (two-fold change) in the PTP/control ratio for a protein so that it could be assigned to proteins whose abundance changed significantly. Essential pore-forming proteins. Since PTP can be opened in mitochondria from different tissues and in media of different ionic strengths, the essential pore-forming components must be determined in all experimental models.

Statistics. Data of in-gel activity staining and channel formation are representative of at least three independent experiments; values of specific activity in bars are means ± S.E.M. (*n* = 6) of three independent experiments. The statistical significance of the differences between the pairs of mean values was evaluated using an ANOVA type 2 (Student–Newman–Keuls) test. The amplitude histograms of conductance summarize the data of three standard records from independent experiments. Values on the curves of current-voltage relationships are means ± S.E.M. (*n* = 3) of three experiments. MS data are presented for three control/PTP pairs of samples; each sample included material from two independent mitochondrial isolations. Data of in-gel activity staining and channel formation are representative of at least three independent experiments. The amplitude histograms of conductance summarize the data of three standard records from independent experiments. Values on the curves of current–voltage relationships are means ± S.E.M. (*n* = 3) of three experiments. MS data are presented for three control/PTP pairs of samples; each sample included material from two independent mitochondrial isolations.

## 3. Results

### 3.1. Effect of PTP Opening on the In-Gel ATPase Activity

**Figure 1:**
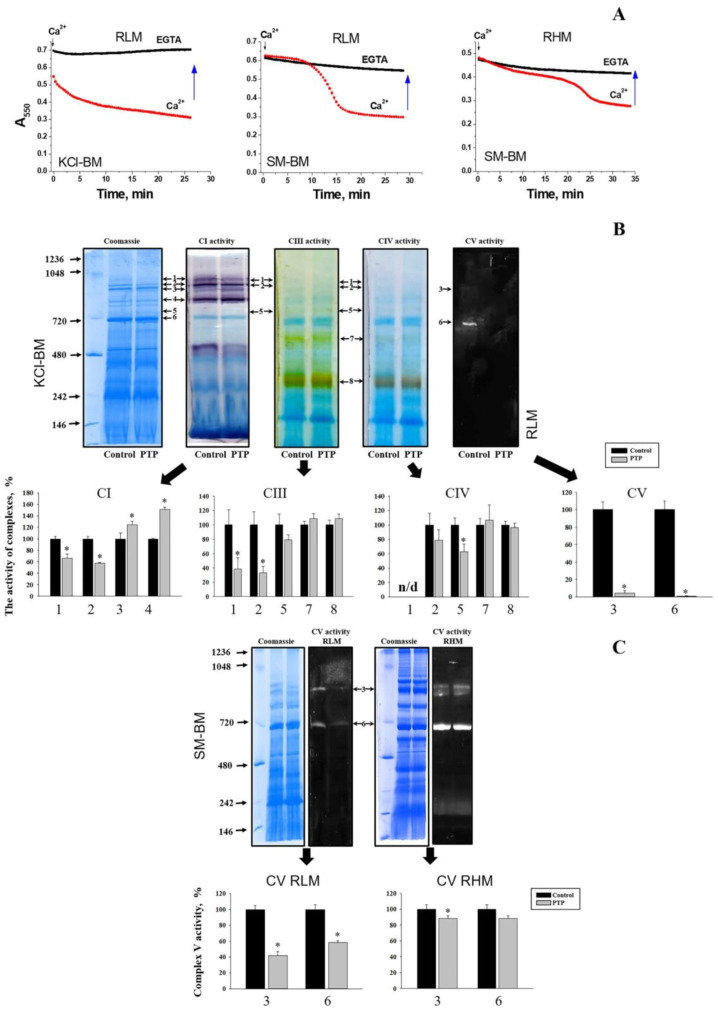
The effect of PTP opening on the in-gel specific activity of F-ATP synthase monomers, dimers, and other OXPHOS supercomplexes from rat liver and heart mitochondria. (**A**) Swelling of RLM and RHM (0.75 mg protein/mL) in KCl- and SM-BM induced by 250 µM Ca^2+^. Arrows show the time when samples were collected for BN-PAGE. (**B**) In-gel staining of CI, CIII, CIV, and CV-specific activity in control and PTP samples from RLM incubated in KCl-BM. Bands designated 1-8 are CI-CIII_2_-CIV_H_ (high molecular weight), CI-CIII_2_-CIV_L_ (low molecular weight), CV_2_ (dimer), CI, CIII_2_-CIV_x_, CV (monomer), CIII_2_, and CIV, respectively. (**C**) In-gel ATPase activity in control and PTP samples from RLM and RHM incubated in SM-BM. Specific activity staining was performed as described in Materials and Methods. All the figures are representative of at least three independent experiments. The numbered bars below show changes in the corresponding specific activities. The asterisk shows significant differences between control and PTP samples (*p* < 0.05).

Mitochondria from different organs and tissues possess a tissue-specific protein composition, which may predetermine the peculiarities in the PTP regulation (sensitivity to Ca^2+^, ROS, and inhibitors) and dynamics. In addition, the properties of the incubation medium may affect the strength of the association of matrix and intermembrane space proteins with lipids and complexes in the inner membrane. Therefore, we first compared the effect of PTP opening on the specific activity of F-ATP synthase dimers and monomers isolated from rat liver and rat heart mitochondria (RLM and RHM) (Figure 1). Preliminarily, RLM were incubated in the presence of 1 mM EGTA (control samples) or 250 µM CaCl_2_ (PTP samples) either in KCl- or sucrose/mannitol-based media (KCl-BM or SM-BM, respectively). RHM were incubated in SM-BM only. Mitochondria were incubated with EGTA or Ca^2+^ until high-amplitude swelling had occurred in the PTP samples. The registration of the swelling indicated that, in each case, 150–250 µM Ca^2+^ was sufficient to induce PTP opening during a 15–35 min incubation (Figure 1A and Figure S1A). PTP opening had a minor effect on the total specific in-gel activities of respiratory complexes I (CI), III (CIII), and IV (CIV): declined the activities of respiratory complexes organized in supercomplexes but increased or insignificantly affected the activities of separate complexes (Figure 1B and Figure S1A) (the figure shows the activity of RLM complexes incubated in KCl-BM). ATPase activity declined in PTP samples from both RLM and RHM, the decrease in KCl-BM being more pronounced (Figure 1B,C). In the presence of CsA, Ca^2+^ at the same concentrations negligibly affected the specific activity of both separate complexes (CI, CIII, and CIV) and supercomplexes and the ATPase activity of CV dimers and monomers (Figure S1B,C). Thus, PTP opening and/or accompanying high-amplitude long-term swelling destabilizes the F-ATP synthase and reduces its in-gel activity after separation.

### 3.2. Channel-Forming Activity of Dimers and Monomers of the F-ATP Synthase from Control and PTP Samples

**Figure 2:**
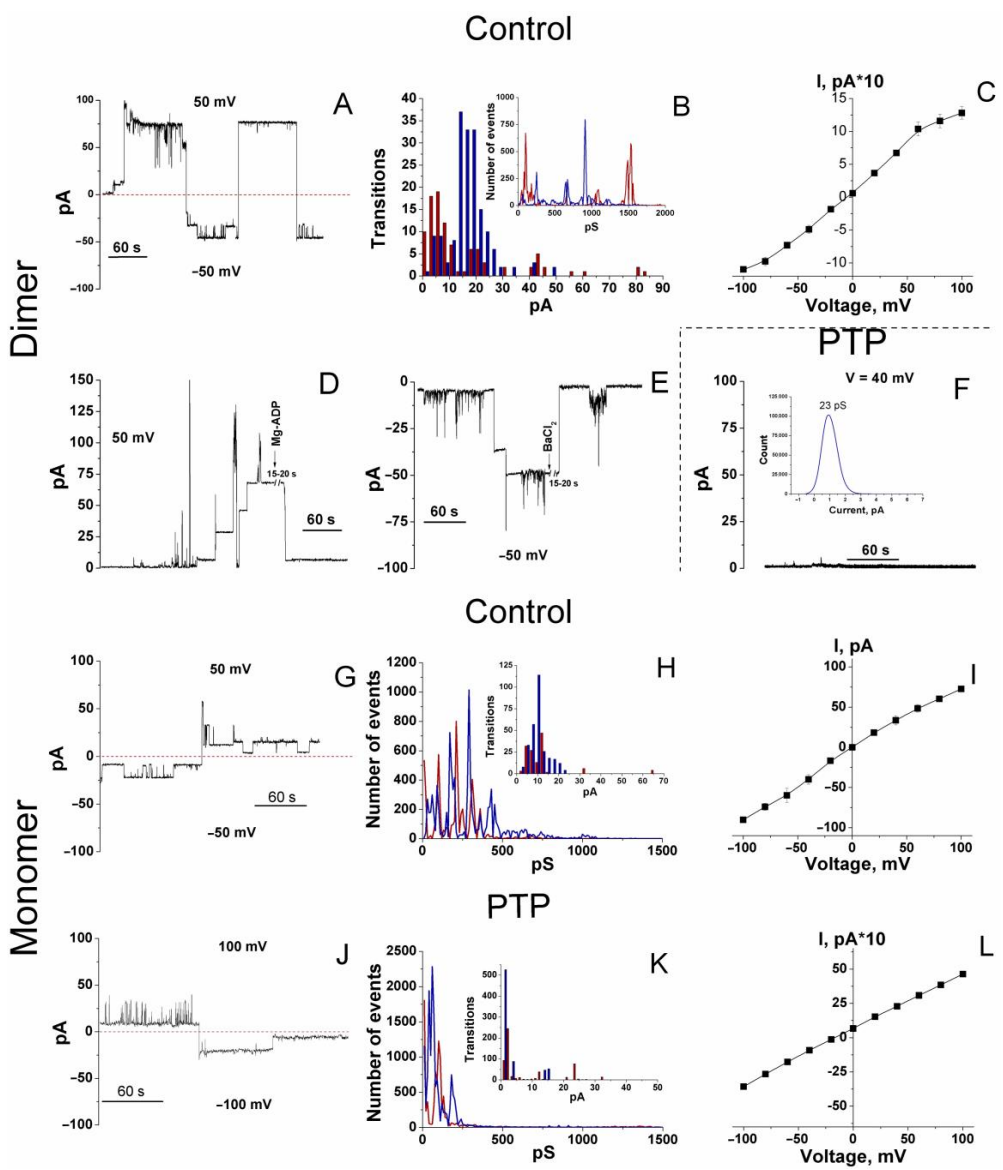
Channels formed in planar lipid membranes via the F-ATP synthase dimers and monomers from control and PTP samples. (**A**,**F**,**G**,**J**) Representative channel activity of dimers from control and PTP samples and monomers from control and PTP samples, respectively. Currents were recorded at indicated positive and negative voltage. Protein eluate (30–90 µL/mL) and calcium (300–900 µM) were added from the trans side of the membrane. Dashed lines show the closed state of the channels. (**B**,**H**,**K**) The corresponding histograms of amplitudes of conductance (pS) and transitions (pA) indicate the probability of channels residing in different conductance states and transiting from one state to another, respectively. For analysis, we took transitions leading to a change in conductance with a duration of at least 200 ms. Red and blue bars and lines correspond to positive and negative voltage, respectively. Each histogram summarizes the data of three records from independent experiments. (**D**,**E**) Effect of 2 mM Mg-ADP and 2 mM BaCl_2_ on the conductance of the dimer from control samples. (**C**,**I**,**L**) Current–voltage relationships were obtained for the control dimer, the control monomer, and the PTP monomer, respectively. Values on the curves are the means ± S.E.M. (*n* = 3) for three independent experiments. The final Ca^2+^ concentration was 300 µM. The interval between the additions of the protein and Ca^2+^ was about 5 min.

Then, we examined whether the alterations in the activity of F-ATP synthase monomers and dimers after the PTP opening are associated with changes in their channel-forming capability. Complexes and associated proteins were eluted from dimer and monomer bands of RLM control and PTP samples incubated in SM-BM, after which they were incorporated in bilayer soybean lecithin membranes (Figure 2). The incorporation of F-ATP synthase dimers from control samples into membranes did not induce any channel activity unless Ca^2+^ (300–900 µM) was added at the same (*trans*) side of membranes. Ca^2+^ evoked a delayed but abrupt formation of large and sustainable (for up to a minute and even more) channels in six experiments out of seven (Figure 2A and Figure S2).

At positive voltage (the sign of the potential on the figures is shown for the *cis* side of the membrane), large channel states and substates were more stable than at negative voltage. However, the overall conductance was similar. An amplitude histogram revealed that the maximum channel conductance at a negative voltage was approximately 1.22 nS. Additionally, the highest probability of the channel(s) residence was in the conductance states of 910, 670, 250, and 60 pS. At positive voltage, the maximum conductance was about 1.53 nS with the highest probability of the channel(s) residence at ~100, 180, 270, 650 pS, and 1.1 nS (Figure 2B, insert). The probability for channels to reside in other conductance states was several orders of magnitude lower. Since eluates from F-ATP synthase bands were used without additional purification, one can suggest that the channel activity observed was due to both the F-ATP synthase and a contaminating low-conductance channel protein(s). Therefore, we assessed the sensitivity of the currents to known inhibitors of PTP/MMC, Ba^2+^ and Mg-ADP. Both inhibitors caused fast and strong inhibition of the high-conductance channel activity of the dimer (Figure 2D,E).

The analysis of the amplitude of transitions between states revealed that, at negative voltage, the most probable transitions were (in the order of decreasing probability) 330/275, 140–150, 85, and, to a lesser extent, ~825 pS; and at positive voltage: ~185, 100, 440, and 870 pS. Large channels formed more readily at a higher voltage (100 mV), while at a lower voltage (50 mV), they were more stable. At middle voltage values, the current–voltage relationship was essentially linear (Figure 2C).

At protein concentrations three times higher, the conductance reached the values of several nS with transitions of ~0.5 and 1 nS, presumably due to the incorporation of several channel complexes (Figure S3).

Against our expectations, incorporating the dimer from PTP samples followed by treatment with Ca^2+^ in increasing concentrations did not cause any channel activity in four out of five experiments (Figure 2F and Figure S4B). In one experiment, we observed sustainable channels with 70 and 100 pS conductance at a negative and positive voltage (100 mV), respectively (Figure S4B).

Thus, eluates from control dimer bands contain entities forming large MMC-like channels (≥300 pS) and small channels (≤250–270 pS). After the Ca^2+^-mediated assembly of the PTP complex, the F-ATP synthase dimer loses some components essential for forming or stabilizing large channel(s), while the contaminating small channel(s) may preserve its activity.

The data on the capability of an F-ATP synthase monomer to form high-conductance channels are controversial [4,10,11]. In our experiments, monomers from control RLM samples formed Ca^2+^-activated channels, however, of lower conductance and stability than dimers (Figure 2G and Figure S5). Stable states and substates were observed both at positive and negative voltage. At negative voltage, the highest probability of the channel residence was in conductance states of 90, 170, 290, and 430 pS; the conductance of ~650 pS and 1.1 nS was much less probable (Figure 2H). The occurrence of transition amplitudes decreased in the range of 190, 150, and 380 pS. At positive voltage, the channels resided in the states of 100, 210, 310, and 360 pS with rare transitions to 660 pS states. Transition amplitudes were 225, 90, and much rarely 625 and 1280 pS. The current–voltage relationship (Figure 2I) was similar to that of a dimer (Figure 2C). PTP/MMC inhibitor Mg^2+^ (2 mM) inhibited high-conductance channel activity with a certain lag period (Figure S5B).

Eluates of monomers from PTP samples demonstrated reduced channel activity compared to control samples (Figure 2J–L and Figure S6A–D). Like channels formed via a PTP dimer, these channels were less stable at high-conductance states and positive voltage. The conductance with the highest probability occurred at 60 and 180 pS (negative voltage) and <30 and 100 pS (positive voltage), though individual peaks of conductance reached 0.8 and even 1.5 nS (Figure 2K). However, the most frequent transitions were of 140 pS (negative voltage) and 240 and 120 pS (positive voltage), indicating, in the latter case, the activation of short-living channels. The current–voltage relationship was essentially linear (Figure 2L).

Thus, eluates from control F-ATP synthase dimers (F-ATP synthase subunits and associated proteins) can form large and sustained MMC-like channels, eluates from control monomers demonstrate the capability of creating channels with reduced conductance and stability, while eluates from PTP dimers and monomers cannot compose stable high-conductance channels. In all samples, low-conductance channel activity was presumably due to contaminated channel-forming proteins.

Hence, Ca^2+^-dependent PTP opening caused substantial alterations in the bands of F-ATP synthase dimers and monomers (i.e., F-ATP synthase subunits and co-migrating proteins), which affect both specific in-gel activity and channel-forming capacity in planar lipid membranes. One can assume that the Ca^2+^-dependent assembly of a PTP complex in mitochondria before the separation of supercomplexes diminishes the number of available PTP-forming blocks: F-ATP synthase subunits and accompanying proteins. Therefore, eluates from the bands of F-ATP synthase monomers and dimers from PTP samples are devoid of the essential components of the PTP complex and incapable of forming high-conductance channels.

### 3.3. Protein Composition of the Bands of the F-ATP Synthase Monomer and Dimer from Control and PTP Samples

**Figure 3:**
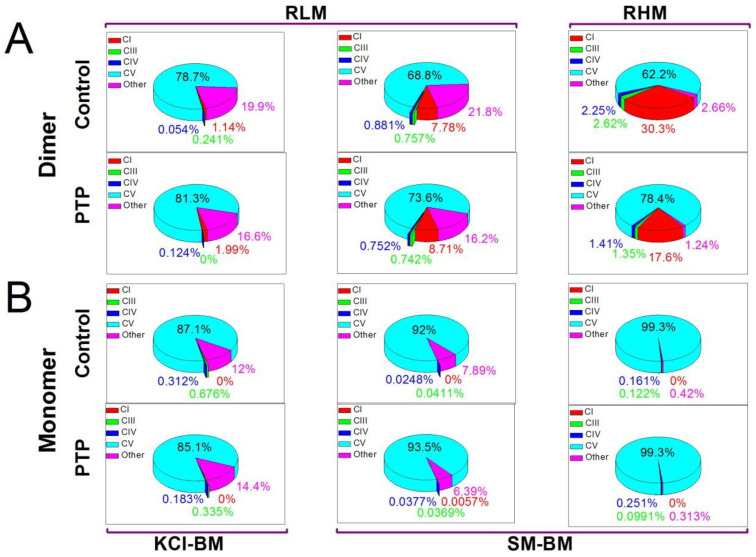
Changes in the protein composition of the bands of the F-ATP synthase monomer and dimer associated with PTP opening. For each variant of mitochondria and incubation medium, dimer (**A**) and monomer (**B**) bands were collected from four BN-PAGE gels of two independent mitochondrial isolations. Each gel contained equal numbers of control and PTP samples. The total content of all peptides determined in a band via MS-MS analysis (100%) (Table S1) was summed from IBAQs of peptides of complexes I (CI), III (CIII), IV (CIV), and V (CV) and non-OXPHOS proteins (other).

Then, we examined whether the PTP-related changes in the specific and channel-forming activities of F-ATP synthase monomers and dimers are associated with alterations in the protein composition of the bands. Dimer and monomer bands in control and PTP samples from RLM (KCl- and SM-BM) and RHM (SM-BM) were analyzed by tandem mass spectrometry (Dataset_S01-Dataset_S06). Since the PTP may be a rare or even a single object in a mitochondrion, all true proteins identified at least by one unique peptide with at least 5% coverage of the protein sequence or by two unique peptides with at least 1% coverage were selected for the analysis of MS spectra (Dataset_S07). The abundance of a protein in a band was determined as the relative intensity-based absolute quantity (rIBAQ): the sum of peak intensities of all unique peptides of a protein (IBAQ) divided by the sum of peak intensities of all unique peptides of all true proteins in the band (Dataset_S07, sheets designated “All”). The pie diagrams show the total content of subunits of complexes I (CI), III (CIII), IV (CIV), and V (CV) and non-OXPHOS mitochondrial proteins (other) in the bands (Figure 3). In addition to CV subunits, dimer bands contained some amounts of subunits of CI, CIII, and CIV, indicating the incomplete separation from the CI–CIII2–CIVx supercomplex, which agrees with the data of WB analysis (Figure S7). Additionally, dimer bands were comprised of 3 (RHM) to 20% (RLM) of non-OXPHOS (other) proteins. In all mitochondrial preparations, PTP samples had a reduced percentage of “other” proteins in dimer bands. The protein composition of monomer bands was much more homogeneous than that of dimer bands: CV subunits amounted to 85–99% of total protein (Figure 3 and Figure S7). The PTP opening caused minor changes in the protein composition of monomer bands from different mitochondrial preparations. Thus, PTP opening caused the elimination of non-OXPHOS proteins from dimer bands with a minor effect on the protein composition of monomer bands.

It should be stressed that F-ATP synthase dimer bands did not contain mitochondrial Ca^2+^- and voltage-activated potassium and chloride channels and exchangers (VDACs, SKCa, IKCa, BKCa, mitoKATP, TASK3, mitoSLO2, mitoHCNs, mitoKv1.3, mitoKv1.5, mitoKv1.7, mitoKv7.4, CLIC1, CLIC4, CLIC 5, MCLCA1, and Letm1) (Dataset_S07) [45,46,47,48]. Other supercomplexes contained some of these proteins (Table S1). Monomers from PTP samples contained a low quantity of mitoKATP (KCNJ8) and VDAC1-3 (Dataset_S07). This disproves the role of mitochondrial channels other than MMC in the observed Ca^2+^-induced MMC-like activity. However, monomer and dimer bands from control and PTP samples contained traces of non-mitochondria channels and transporters: inward rectifier potassium channel 2, sarcoplasmic/endoplasmic reticulum calcium ATPase 1, and transmembrane channel-like protein 8, which, theoretically, may be responsible for low-conductance channel activity.

In accordance with the data of MS-MS analysis, the intensity of signals from some proteins in dimer and monomer bands from control and PTP samples differed tens of times. To identify proteins that could be essential for channel-forming activity, we selected all proteins whose quantity unidirectionally changed “all or nothing” and by two or more times in all pairs of control-PTP (Dataset_S07, sheets designated “big difference”). The number of proteins meeting these criteria was few, with eight OXPHOS complex subunits and two non-OXPHOS proteins (Table 1). The content of three (NADH:ubiquinone oxidoreductase subunits A7 and A13 and F-ATP synthase subunit e) and two proteins (cytochrome b-c1 complex subunit Rieske and very long-chain specific acyl-CoA dehydrogenase) increased in dimer and monomer bands, respectively (at least in part, this can be connected with the partial destruction of the CI–CIII2–CIVx supercomplex (Figure 1) due to the PTP-dependent inactivation of complex I [49]). The level of five proteins (cytochrome b-c1 complex subunits 1 and 2, cytochrome c oxidase subunit 5A, methylmalonate–semialdehyde dehydrogenase (acylating) (Aldh6a1), and prohibitin 2 (Phb2)) and one protein (Aldh6a1) decreased in dimer and monomer bands, respectively (Phb2 was not detected in monomer bands from both control and PTP samples). Hence, the PTP-related decrease in the channel-forming activity of dimers and monomers correlates with a loss or absence of two proteins only: Aldh6a1 and Phb2. The level of the Phb2 partner Phb did not change so drastically, thus considerably reducing the Phb2/Phb ratio (Table 1), which indicates the destruction of the Phb-Phb2 complex. These data suggest that non-OXPHOS proteins Aldh6a1 and prohibitin(s) may be essential for the formation and stability of MMC-like channels.

## 4. Discussion

The data presented disprove our initial hypothesis that the preliminary Ca^2+^-dependent assembly of the PTP/MMC complex from F-ATP synthase and other mitochondrial proteins would increase the channel activity of isolated F-ATP synthase dimers and monomers. Though PTP opening changed the protein composition of the F-ATP synthase monomer and dimer bands (Figure 3, Datasets_S01–S07) and, in accordance with recent observations, reduced the F-ATP synthase stability and/or in-gel activity after separation (Figure 1) [50,51], it, however, drastically inhibited the channel activity of the eluted F-ATP synthase (Figure 2). Nevertheless, our main idea that proteins not included in the F-ATP synthase complex could participate in the PTP/MMC formation and stabilization is, presumably, correct.

Recent protein knockdown- and knockout-based studies revealed the role of the F-ATP synthase complex and its different subunits in the PTP/MMC formation and stability [5,6,7,8,12,18,31,32,33,34,52,53]. In the studies, subunits c plus δ [8,34], c [5,7,33,52], DAPIT (k), e, f, 6.8PL (J) [8], g [8,31], ATP6 (a) [33,53], ATP8 (A6L) [33,53], b [12,31], and OSCP [12,31] were deleted; the levels of subunits f [18,32], c, and g [18] were decreased via siRNA and shRNA. In addition, the level of subunits ATP6 (a), ATP8 (A6L), c, d, e, f(1/2), F6, g, 6.8PL (J), DAPIT (k), OSCP, γ, and δ, declined to a different extent due to the deletion of other F-ATP synthase subunits [8,12,31,33]. Although the results and their interpretations are somewhat controversial (apparently, this is due to the different parameters used for the registration of PTP opening: calcium retention capacity, quenching of calcein fluorescence, mitochondrial swelling in KCl- and sucrose-based medium, swelling/shrinkage in the presence of PEGs of different size, and channel activity in mitoplasts, bilayer lipid membranes, and vesicles [5,6,7,8,12,18,31,32,33,52,53]), several conclusions about the molecular nature of PTP/MMC can be made. First, subunits g, f, and c are, perhaps, of critical importance for the PTP/MMC formation [8,31,50]. In fact, the deletion of subunit g completely suppresses the MMC-like channel activity [31] and inhibits the swelling in KCl-BM [8,31]. Similarly, the deletion or suppression of subunit f inhibits the swelling in KCl- and sucrose-BM [8,31]. The deletion of subunit c weakly affects the net Co^2+^ accumulation upon prolonged incubation [8,33] but inhibits it in short-term experiments [5,52]; in addition, it inhibits the swelling in sucrose medium [8] and reduces the size of MMC channels [7,50]. (The suppression or deletion of all these subunits does not affect the mitochondrial calcium retention capacity [8,18,33] because the release of calcium does not require the formation of high-conductance channels). Second, an intact F-ATP synthase monomer or dimer is presumably unnecessary for assembling a PTP complex [8,10,11]. Third, PTP opening is a relatively rare event and does not require the involvement of all critical F-ATP synthase subunits in a mitochondrion [37]. Indeed, even a drastic decrease in the level of some ATP subunits was insufficient for the suppression of channel activity, and their complete removal was required. At the same time, the incomplete removal of some subunits decreased the integral index of high-amplitude swelling of mitochondria in a sucrose-based medium or the presence of large PEGs [8,12,31,33].

Our data agree with the view that F-ATP synthase subunits are the core components of a PTP, while entire complexes are not. Indeed, only the eluates from the bands of F-ATP synthase dimers and monomers, but not from bands of CI-CIII_2_-CIV_x_ and CIII_2_-CIV_x_ supercomplexes (Figure S8), demonstrated high-conductance channel activity in planar membranes. Further, F-ATP synthase dimers and monomers from PTP samples demonstrated a dramatically reduced channel activity (Figure 2) despite the fact that they possessed the same set of subunits as control dimers and monomers (Dataset_S7). Taking into account that assembled and active F-ATP synthase is avoidable for PTP/MMC formation [8,10,11], partial inactivation of F-ATP synthase during the PTP opening and BN-PAGE (Figure 1) cannot explain the decrease in channel-forming activity in monomers and dimers from PTP samples (Figure 2) since these bands from both control and PTP samples contain the same set of the complex subunits (Datasets_S01–S07). (Making this conclusion, we assume that the efficiency of protein elution from both types of samples is similar).

Nevertheless, the changes in the protein composition of dimer and monomer bands occurred and covered some OXPHOS subunits and a few non-OXPHOS proteins (Table 1). Dimer and monomer bands accumulated complex I and complex III plus IV subunits, respectively, which, most likely, was connected with the partial destruction of the CI-CIII_2_-CIV_x_ supercomplex (Figure 1). (The substantial inactivation of complex I due to PTP opening was previously reported [49].) What is more important, the decline in the level of Aldh6a1 (dimer and monomer bands) and Phb2 (dimer bands; monomer bands lacked prohibitins) correlated with the decrease in the MMC-like channel activity of eluates from PTP samples (Figure 2, Table 1). These data indicate that minor F-ATP synthase-associated proteins are, presumably, of critical importance for PTP/MMC complex formation, which explains the relative rareness of PTP as an object in a mitochondrion [37]. It is remarkable that purified dimers and monomers from bovine and porcine hearts [2,4,10] formed comparatively less stable channels than dimers of F-ATP synthase from RLM (Figure 2), which contained a large portion of attendant proteins (Figure 3, Dataset_S07). Channels formed by dimers from RLM were similar in stability to MMC channels in rat liver mitoplasts [39,40,41] and mitoplasts from HeLa cells [31]. In addition, recent crystallographic studies of the structure of highly purified F-ATP synthase in the presence and absence of Ca^2+^ demonstrated that conditions favoring PTP formation cause a disorganization of the interactions of membrane-immersed subunits, including the collapse of the c-subunit ring [51]. Thus, it seems likely that it is the level of minor F-ATP synthase-associated proteins but not of F-ATP synthase subunits that limits the abundance of the PTP complex in a mitochondrion and, therefore, maximal purification of F-ATP synthase dimers and monomers in the studies of channel activity [2,4,10] is a dead end in research. On the other hand, Aldh6a1 and Phb2 are obviously insufficient for MMC-like channel formation since eluates from the bands of CI-CIII_2_-CIV_x_ and CIII_2_-CIV_x_ supercomplexes did not demonstrate high-conductance channel activity in planar membranes (Figure S8), although the bands contained Aldh6a1 and Phb2 (Datasets_S08–S10, Table S2).

**Figure 4:**
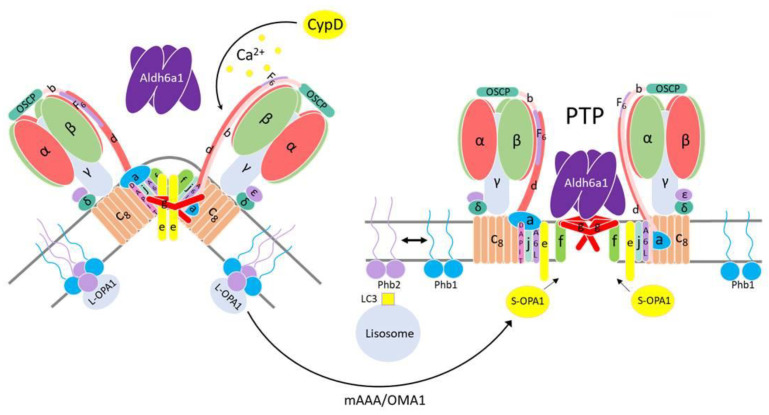
A proposed mechanism for the participation of Aldh6a1 and prohibitins in the PTP formation by subunits of a F-ATP synthase dimer. For explanations, see the text. mAAA and OMA1 are the ATP-dependent metalloendopeptidases of the inner mitochondrial membrane, involved in the proteolytic activation of optic atrophy protein 1 (OPA1); L-OPA1 and S-OPA1 are the full-length and truncated OPA1 forms, respectively.

At the present research stage, only a speculative model of the PTP/MMC channel and its formation mechanism can be proposed. Along with the membrane F-ATP synthase subunits g, f, and, probably, c, a stable PTP complex may comprise a matrix Aldh6a1 tetramer. In order to explain the absence of Aldh6a1 and channel activity in monomers and dimers from PTP samples, one may assume that a stable PTP complex (Aldh6a1_4_-g_n_-f_n_-subunit c(?)_n_) loosely associates with an F-ATP synthase complex and migrates separately in BN-PAGE gels. For this to happen, Ca^2+^ should weaken the interaction of the F-ATP synthase subunits g and f with their partner subunits a, b, and e [54] and enhance the interaction with proximal available Aldh6a1 (Figure 4). (The separation of a membrane-immersed subcomplex from the matrix-oriented soluble subcomplex during the PTP opening was confirmed in recent works [50,51]). If this assumption is correct, the molecular mass of the PTP complex should be at least 270 kDa and hardly exceed 500 kDa.

The data on the involvement of Aldh6a1 in the regulation of PTP opening or cell death are scarce. It was shown that the expression of Aldh6a1 is reduced ten times in different hepatocellular carcinomas [55], while hepatic carcinoma mitochondria are extremely resistant to calcium [56]. The restoration of Aldh6a1 expression induced the loss of mitochondrial potential and cell death [55]. Matrix NADH inhibited both Aldh6a1 (Ki 3.1 µM) [57] and PTP opening [58]. The level of Aldh6a1 in the liver and kidney was found to be higher than in the heart and the brain [59], which correlates with the ability of mitochondria from these tissues to swell in a PTP-dependent way (Figure 1) [59,60]. In addition, tyrosine nitration of Aldh6a1 is enhanced in rat kidney mitochondria upon the development of diabetes mellitus Type 1 [61] and acute septic damage [62] and in heart mitochondria in aging [63]. The antagonists of peroxynitrite production suppressed the nitration of Aldh6a1, damage to the kidney, and animal mortality [62]. On the other hand, preconditioning increased the level of Aldh6a1 and F-ATP synthase β subunit in heart mitochondria as well as their resistance to ischemia/reperfusion [64], though the association of Aldh6a1 with F-ATP synthase was not explored.

By contrast, the analysis of publications argues against the direct involvement of Phb2 in the PTP/MMC complex formation. First, the Phb–Phb2 complex is essential for mitochondrial resistance to PTP opening and cell resistance to apoptotic stimuli [65,66,67]. Second, the Phb–Phb2 complex increases the stability of mitochondrial supercomplexes CI–CIII2–CIV_x_ and CIII2–CIV_n_ [68,69] and, most probably, F-ATP synthase oligomers through the stabilization of dynamin-related GTPase OPA1 [70,71,72]. Third, the PTP-dependent collapse of the membrane potential triggers mitophagy, which requires the Phb–Phb2 complex destruction and Phb2 binding with LC3 [73,74]. Therefore, one could assume that the destruction of the Phb–Phb2 complex associated with the F-ATP synthase dimer causes a destabilization of the latter and facilitates PTP complex formation [11] (Figure 4). Concomitantly, liberated Phb2 becomes a receptor that activates the elimination of a damaged mitochondrion.

To conclude, here we described the effect of the preliminary PTP opening in mitochondria on the protein composition of the F-ATP synthase dimer and monomer BN-PAGE bands and the high-conductance channel-forming ability of eluates from these bands. The data obtained indicate that proteins neighboring F-ATP synthase, namely, Aldh6a1 and Phb2, may be important players in the PTP/MMC formation and stabilization.

## Figures and Tables

**Table 1:** Proteins in the bands of monomers and dimers, the level of which changed unidirectionally after the PTP opening.

Protein	PTP/Control rIBAQ Ratio		
Dimer	RHM	RLM SM-BM	RLM KCl-BM
NADH:ubiquinone oxidoreductase subunit A7	7.25	1.57	0.008/ND
NADH:ubiquinone oxidoreductase subunit A13	1.26	4.51	0.038/ND
ATP synthase subunit e mitochondrial	0.322/ND	1.34	0.315/ND
Cytochrome b-c1 complex subunit 1	0.13	0.75	ND/0.0044
Cytochrome b-c1 complex subunit 2	0.49	0.75	ND/0.234
Cytochrome c oxidase subunit 5A	0.19	0.58	ND/0.003
Methylmalonate–semialdehyde dehydrogenase [acylating]	ND/0.118	0.028	0.029
Prohibitin-2	0.18	0.51	0.013
Prohibitin	0.507	0.609	0.834
Monomer			
Cytochrome b-c1 complex subunit Rieske	2.10	4.21	1.89
Very long-chain specific acyl-CoA dehydrogenase	0.007/ND	1.43	3.86
Methylmalonate–semialdehyde dehydrogenase [acylating]	ND/ND	0.40	0.348
Prohibitin-2 *	ND/ND	ND/ND	ND/ND
Prohibitin *	ND/ND	ND/ND	ND/ND

Notes: ND—not detected; *—absent in all monomer samples. In pairs where a protein was absent (ND) either in a control or a PTP sample, the rIBAQ value is given for the present protein.

## Data Availability

The data presented in this study are available in the article supplementary material “Supplementary Information for Mitochondrial F-ATP synthase-co-migrating proteins and Ca^2+^-dependent formation of large channels”, including Figures S1–S8, Tables S1 and S2, and in Datasets S1–S10.

## Supplementary Materials

The following supporting information can be downloaded at https://www.mdpi.com/article/10.3390/cells12192414/s1, title “Supplementary Information for Mitochondrial F-ATP synthase-co-migrating proteins and Ca^2+^-dependent formation of large channels” including Figure S1, title “Effect of PTP opening on the in-gel specific activity of F-ATP synthase monomers and dimers and other OXPHOS (super)complexes from rat liver and heart mitochondria”; Figure S2, title “Representative channel-forming activity of mitochondrial F-ATP synthase dimer from control samples in the media supplemented with 200 (A) and 150 mM KCl (B)”; Figure S3, title “Multiple channel activity of mitochondrial F-ATP synthase dimer from control samples (A) and the corresponding amplitude histogram of conductance (B)”; Figure S4, title “F-ATP synthase dimer from PTP samples with (A) and without channel activity (B)”; Figure S5, title “Representative channel activity of F-ATP synthase monomer from control samples. Currents were recorded at 100/50 mV (+/-) (A) and at 100 mV (-) (B)”; Figure S6, title “Variations in the channel activity of F-ATP synthase monomer from PTP samples”; Figure S7, title “Contamination of F-ATP synthase monomer and dimer bands from control and PTP samples by OXPHOS proteins”; Figure S8, title “Representative channel-forming activity of mitochondrial supercomplexes from control and mPTP samples eluted from the bands enriched with high (A,B) and low (C,D) molecular weight supercomplexes CI-CIII_2_-CIV_x_ and with CIII_2_-CIV_x_ supercomplex (E,F)”; Table S1, title “Mitochondrial ion channels and exchangers associated with (super)complexes”; Table S2, title, “Changes in the level of prohibitin, prohibitin 2, and methylmalonate–semialdehyde dehydrogenase in the bands of mitochondrial (super)complexes after the PTP opening”, Legends for Datasets S1 to S10. Other Supplementary Materials for this manuscript include the following: Datasets S1, title “Mass-spectrometry data of protein content of F-ATP synthase monomer and dimer bands from control samples of RHM”; Datasets S2, title “Mass-spectrometry data of protein content of F-ATP synthase monomer and dimer bands from PTP samples of RHM”; Dataset S3, title “Mass-spectrometry data of protein content of F-ATP synthase monomer and dimer bands from Control samples of RLM incubated in KCl-BM”; Dataset S4, title “Mass-spectrometry data of protein content of F-ATP synthase monomer and dimer bands from PTP samples of RLM incubated in KCl-BM”; Dataset S5, title “Mass-spectrometry data of protein content of F-ATP synthase monomer and dimer bands from Control samples of RLM incubated in SM-BM”; Dataset S6, title “Mass-spectrometry data of protein content of F-ATP synthase monomer and dimer bands from PTP samples of RLM incubated in SM-BM”; Dataset S7, title “Comparison of protein composition of F-ATP synthase dimers and monomers from control and PTP samples”; Dataset S8, title “Protein composition of high- (H) and low-molecular weight (L) CI-CIII_2_-CIV_x_ supercomplexes and CIII_2_-CIV_x_ supercomplex from control and PTP samples isolated from RHM”; Dataset S9, title “Protein composition of high- (H) and low-molecular weight (L) CI-CIII_2_-CIV_x_ supercomplexes and CIII_2_-CIV_x_ supercomplex from control and PTP samples isolated from RLM incubated in SM-BM”; Dataset S10, title “Protein composition of high- (H) and low-molecular weight (L) CI-CIII_2_-CIV_x_ supercomplexes and CIII_2_-CIV_x_ supercomplex from control and PTP samples isolated from RLM incubated in KCl-BM”.
