# Supplementary material for: Mitochondrial F-ATP Synthase Co-Migrating Proteins and Ca2+-Dependent Formation of Large Channels
Source: Cells. 2023 Oct 7;12(19):2414. doi: 10.3390/cells12192414 (PMC10572550; doi:10.3390/cells12192414)
Supplement: Supplementary file 1 [file cells-12-02414-s001.zip › cells-2506926-supplementary.pdf]

Supplementary Information for

Mitochondrial F-ATP synthase-co-migrating proteins and Ca^{2+}-dependent formation of large channels.

Anna B. Nikiforova, Yulia L. Baburina, Marina P. Borisova, Alexey K. Surin, Ekaterina S. Kharechkina, Olga V. Krestinina, Maria Y. Suvorina, Svetlana A. Kruglova, and Alexey G. Kruglov

Alexey G. Kruglov
Email: krugalex@rambler.ru

This PDF file includes:

Figures S1 to S8
Tables S1 to S2
Legends for Datasets S1 to S10

Other supplementary materials for this manuscript include the following:

Datasets S1 to S10

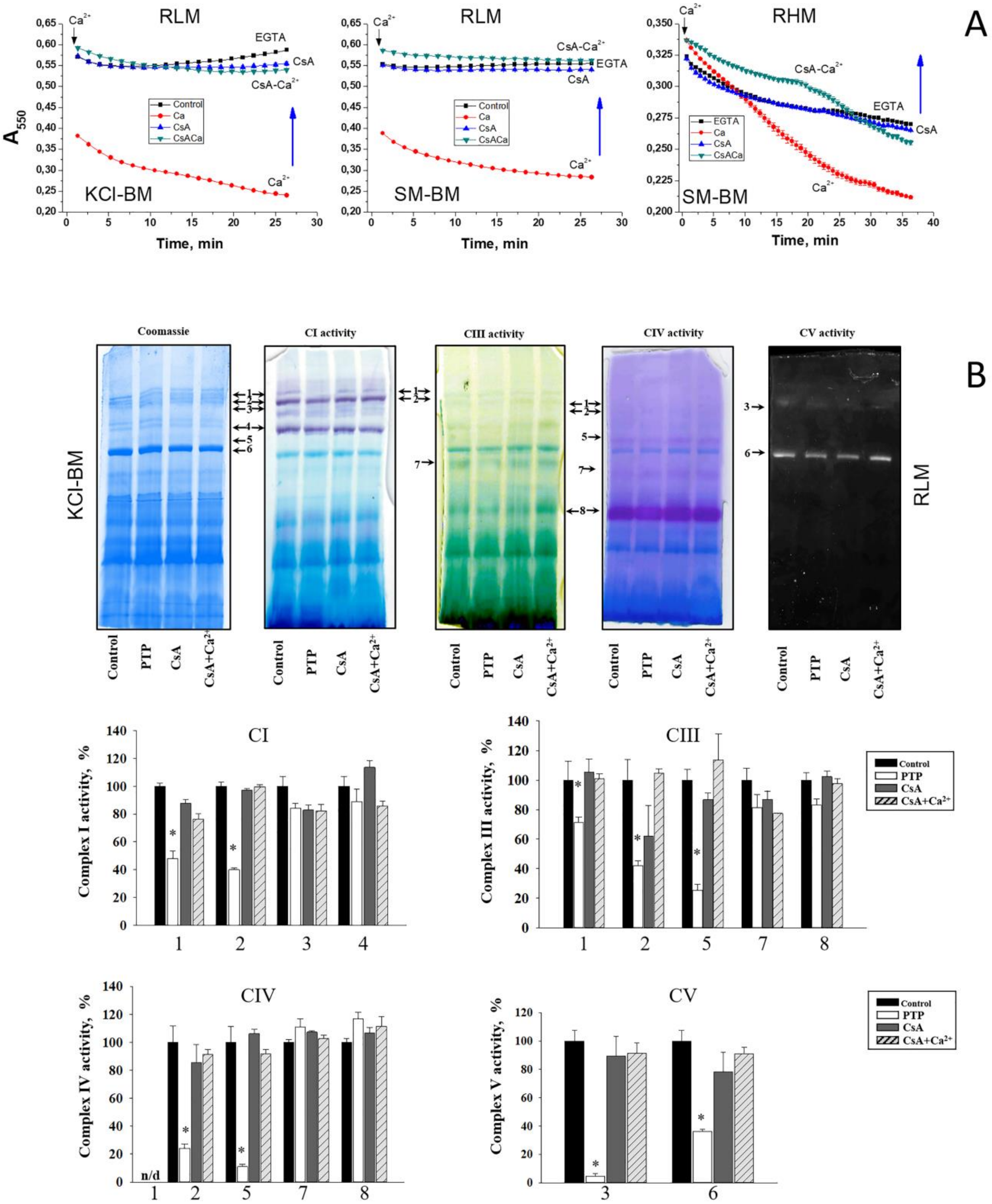
A
RLM
KCl-BM
RLM
SM-BM
RHM
SM-BM
A_{550}
Time, min
Control
Ca
CsA
CsACa
EGTA
CsA-Ca2+
Ca2+
B
KCl-BM
RLM
Coomassie
CI activity
CIII activity
CIV activity
CV activity
Control
PTP
CsA
CsA+Ca2+
CI
Complex I activity, %
CIII
Complex III activity, %
CIV
Complex IV activity, %
CV
Complex V activity, %
n/d

C

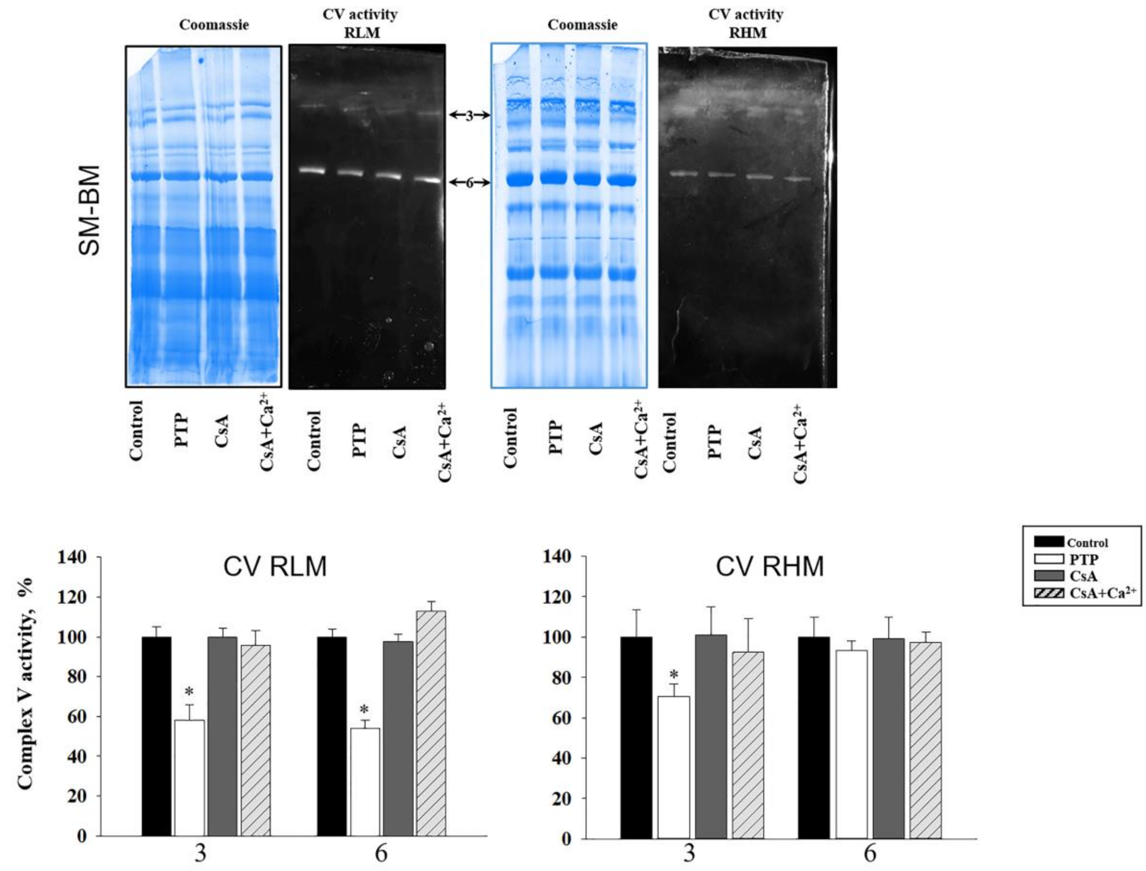

Figure S1. Effect of PTP opening on the in-gel specific activity of F-ATP synthase monomers and dimers and other OXPHOS (super)complexes from rat liver and heart mitochondria. A. Swelling of RLM and RHM (0.75 mg protein/ml) in KCl- and SM-BM induced by 150 µM Ca^{2+} in the presence and absence of 1 µM cyclosporine A (CsA). Control samples contained 1 mM EGTA. Blue arrows show the time when samples were collected for BN-PAGE. Points of the curves are the means ± S.E.M of 12 technical replicates (n = 12). Data of two representative experiments of four identical are presented. B. In-gel staining of CI, CIII, CIV, and CV specific activity in the RLM samples obtained as shown in the left panel A (KCl-BM). Bands designated 1-8 are CI-CIII$_2$-CIV$_H$ (high molecular weight), CI-CIII$_2$-CIV$_L$ (low molecular weight), CV$_2$ (dimer), CI, CIII$_2$-CIV$_x$, CV (monomer), CIII$_2$, and CIV, respectively. C. In-gel ATPase activity in the RLM and RHM samples obtained as shown in the central and right panels A, respectively (SM-BM). Specific activity staining was performed as described in Materials and Methods. Gel data are representative of three independent experiments. Numbered bars below show the changes in the specific activities in the corresponding bands. Values in the columns are the means ± S.E.M of 3-6 independent experiments; n = 6 (control and PTP samples) and n = 3 (CsA and CsA-Ca^{2+} samples). The asterisk shows significant differences with control samples ($p < 0.05$).

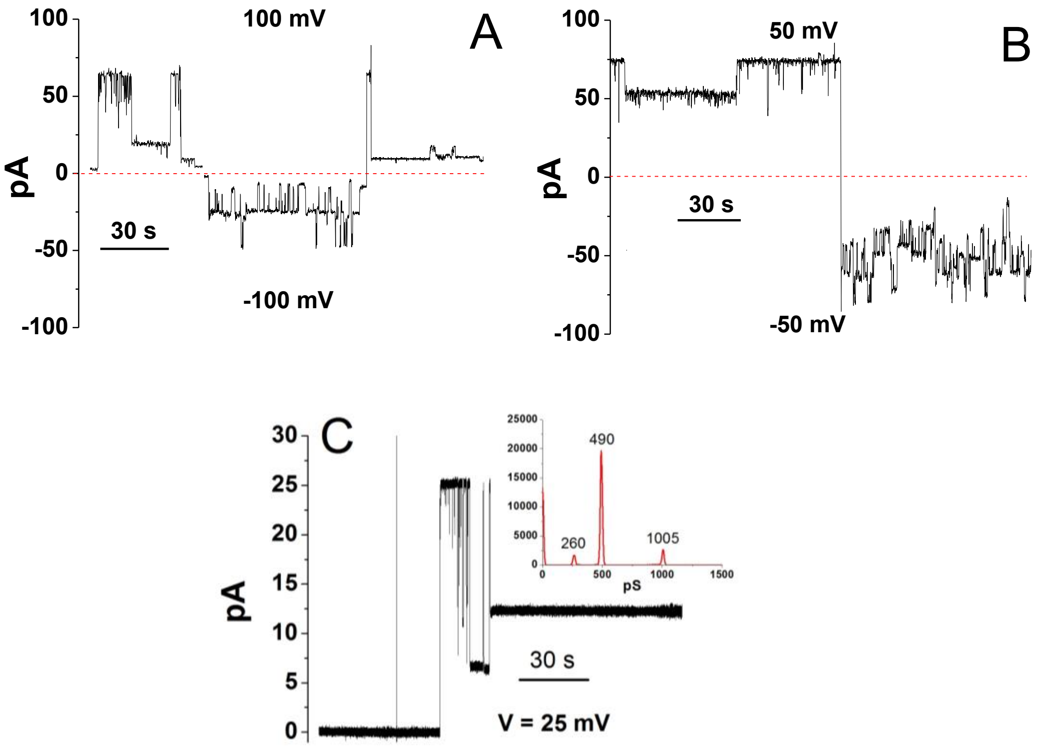

Figure S2. Representative channel-forming activity of mitochondrial F-ATP synthase dimer from control samples in the media supplemented with 200 (A and B) and 150 mM KCl (C). Currents were recorded at 100 (+/-) (A), 50 mV (+/-) (B) and at 25 mV (+) (C). Protein eluates (90 µl/ml) and $CaCl_2$ (300 µM) were added from the *trans* side of the membrane. Insert is the corresponding amplitude histogram of conductance.

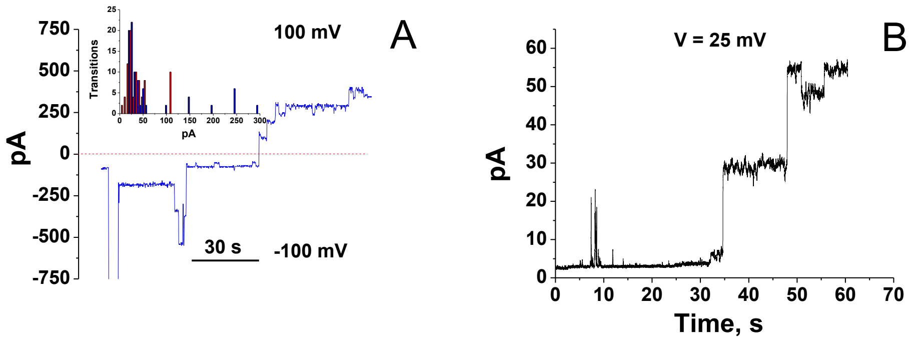

Figure S3. Multiple channel activity created by eluates of mitochondrial F-ATP synthase dimer from control samples. Currents were recorded at 100 **(+/-)** mV (A) and 25 mV (B) in 200 and 150 mM KCl, respectively. Insert in A shows the histogram of transition amplitudes. Protein concentrations in eluates were three times higher than in standard experiments. Protein eluates (90 µl/ml) and $CaCl_2$ (300 (A) and 600 µM (B)) were added from the *trans* side of the membrane.

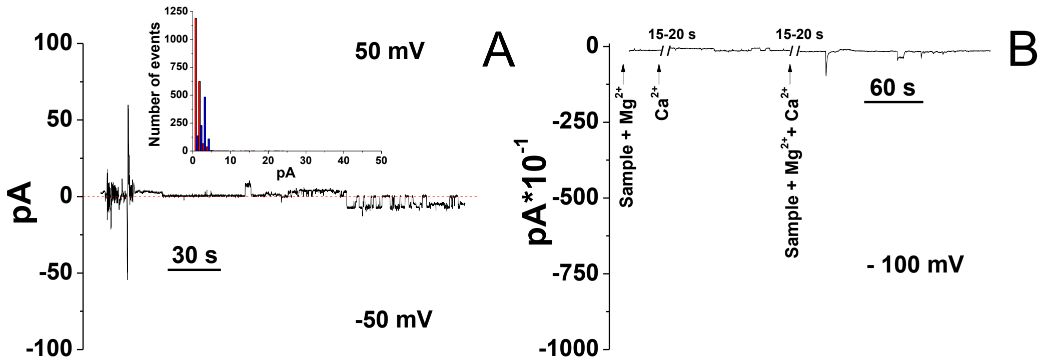

Figure S4. F-ATP synthase dimer from PTP samples with (A) and without channel activity (B). Currents were recorded at 50 **(+/-)** mV (A) and at -100 mV (B). A. Protein eluates (90 µl/ml) and $CaCl_2$ (300 µM) were added from the *trans* side of the membrane. Inserts are the corresponding amplitude histograms of conductance. B. Protein eluates was added in pulses of 45 µl/ml, which contained 315 µM Mg^{2+} (final concentration); $CaCl_2$ was added in pulses of 500 µM (final concentration). The spaces show the time spent adding reagents and mixing.

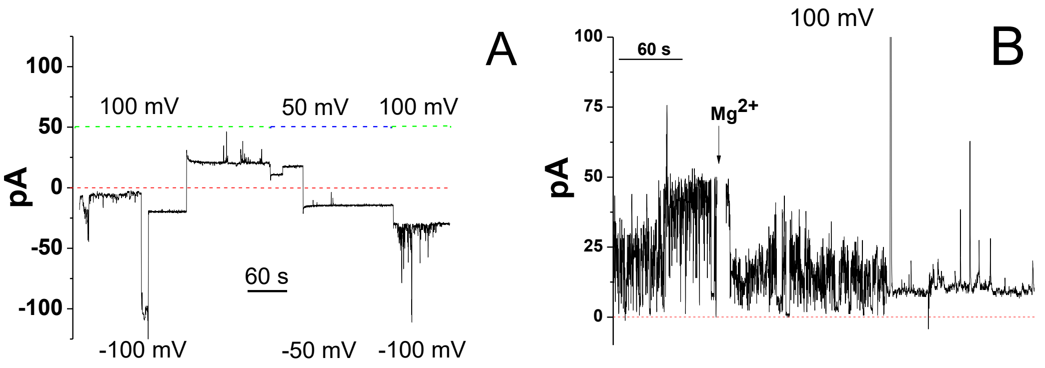

Figure S5. Representative channel activity of F-ATP synthase monomer from control samples. Currents were recorded at 100/50 **(+/-)** mV (A) and at 100 mV (B). Red dashed line shows the closed state of the channel. Green and blue dashed lines indicate the time when 100 and 50 mV voltage was applied, respectively. Protein eluates (30 µl/ml(A) and 50 µl/ml(B)), $CaCl_2$ (300 µM(A) and 1mM(B)) and $MgCl_2$ (2mM(B)) were added from the *trans* side of the membrane.

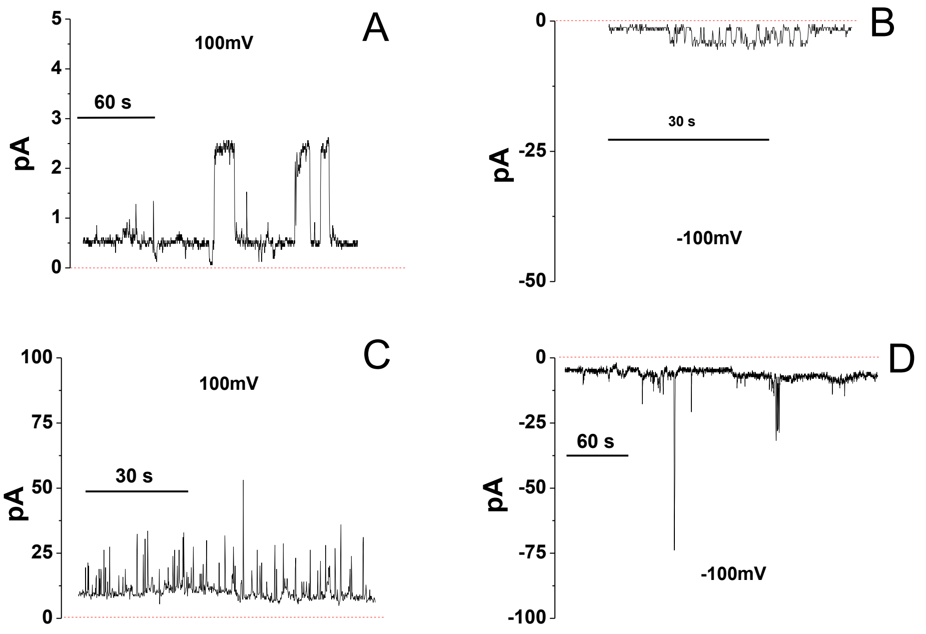

Figure S6. Variations in the channel activity of F-ATP synthase monomer from PTP samples. Currents were recorded at 100 **(+/-)**. Protein eluates (30 µl/ml) and $CaCl_2$ (300 µM) were added from the *trans* side of the membrane. Red dashed lines indicate the closed state of the channel.

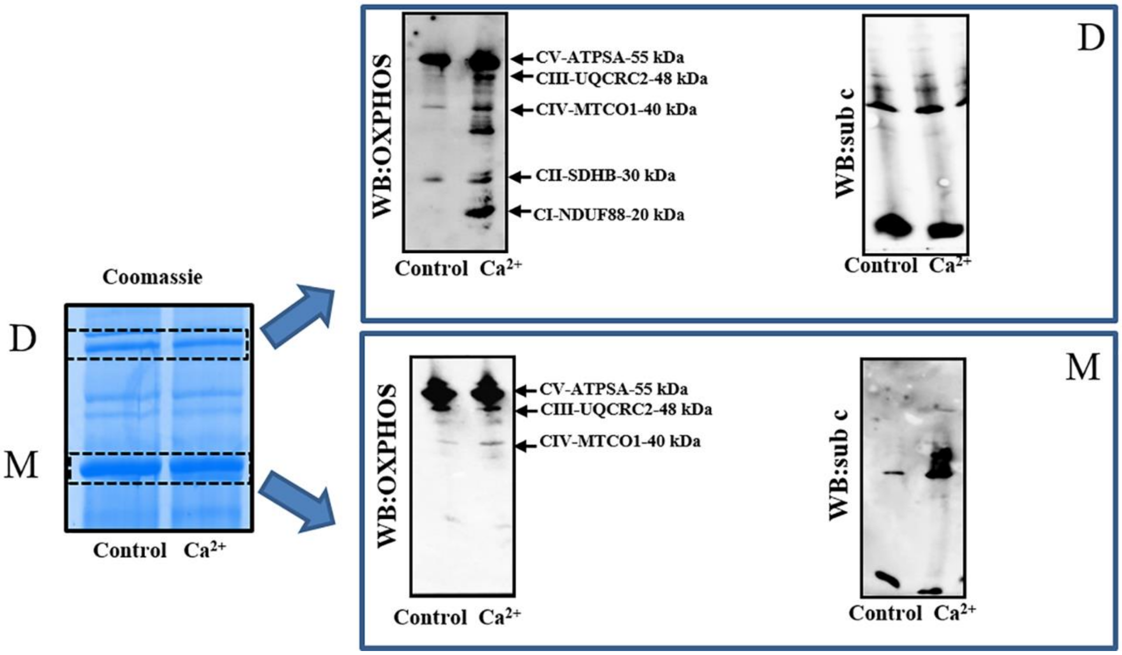

Figure S7. Contamination of F-ATP synthase monomer and dimer bands from control and PTP samples by OXPHOS proteins. F-ATP synthase monomer (M) and dimer bands (D) from RLM control and PTP samples (Ca^{2+}) incubated in SM-BM were subjected to SDS-PAGE and immunoblotting for OXPHOS subunits Ndufb8 (CI), Sdhb (CII), Uqcrc2(CIII), Mt-co1(CIV), Atp5a (CV) (WB: OXPHOS), and ATP5MC1 (CV) (WB: sub c). All the figures are representative of at least three independent experiments.

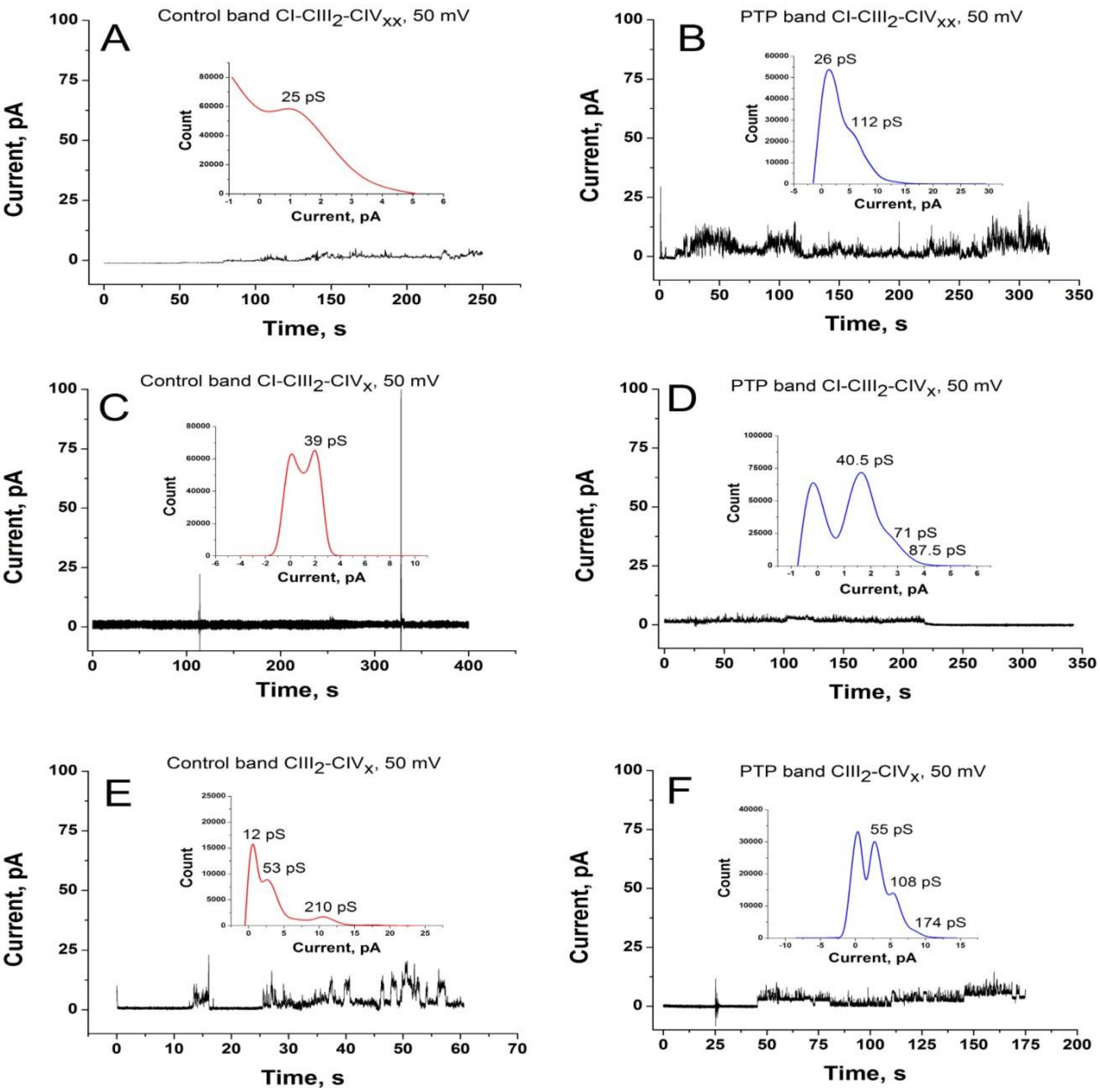

Figure S8. Representative channel-forming activity of mitochondrial supercomplexes from control and mPTP samples eluted from the bands enriched with high (A and B) and low (C and D) molecular weight supercomplexes CI-CIII$_2$-CIV$_x$ and with CIII$_2$-CIV$_x$ supercomplex (E and F). The elution buffer for CI-CIII$_2$-CIV$_x$ supercomplexes was the same as for F-ATP synthase dimers and monomers except ATP was replaced by 5 mM NADH. Currents were recorded at +50 mV. Protein eluates (90 µl/ml) and $CaCl_2$ (300 µM) were added from the *trans* side of the membrane. Inserts are the corresponding amplitude histograms of conductance.

Table S1. Mitochondrial ion channels and exchangers associated with (super)complexes. Samples were obtained from the RHM and RLM incubated in SM-BM and RLM incubated in KCl-BM in the absence (Control samples) and presence of Ca^{2+} (PTP samples). Data from the corresponding raw MS datasets were extracted by PEAKS Studio 7.5/XPro. Designations: asterisk shows the presence of a protein at the trace quantity; H and L indexes at the $CI\text{-}CIII_2\text{-}CIV_x$ supercomplex indicate high and low molecular weight of the latter. Clic1, chloride intracellular channel protein 1; Clic4, chloride intracellular channel protein 4 (intracellular chloride ion channel protein p64H1); Letm1, mitochondrial proton/calcium exchanger protein (leucine zipper-EF-hand-containing transmembrane protein 1); Letmd1, LETM1 domain-containing 1; Letm2, LETM1 domain-containing protein LETM2, mitochondrial; Kcnj8, ATP-sensitive inward rectifier potassium channel 8 (inward rectifier K(+) channel Kir6.1); Slc8a2, sodium/calcium exchanger 2; Slc8a3, sodium/calcium exchanger 3; Slc8b1, mitochondrial sodium/calcium exchanger protein (solute carrier family 24 member 6); Vdac 1-3, voltage-dependent anion-selective channel protein 1-3.

**(Super)complex**	**RHM**		**RLM SM-BM**		**RLM KCl-BM**	
	Control	PTP	Control	PTP	Control	PTP
$CI\text{-}CIII_2\text{-}CIV_x$ (H)			Letm2<br>Slc8b1	Letmd1<br>Slc8b1		Letm2
$CI\text{-}CIII_2\text{-}CIV_x$ (L)				Clic4		
CV_2			Slc8b1*	Slc8b1*		
CI		Vdac 1	Slc8a3			Clic1<br>Slc8b1
$CIII_2\text{-}CIV_x$	Letm1*			Vdac 1		Vdac 1
CV		Vdac 3<br>Kcnj8*	Vdac 3	Slc8a2*<br>Slc8b1*		Letm1<br>Vdac 1<br>Vdac 2<br>Vdac 3*

Table S2. Changes in the level of prohibitin, prohibitin 2, and methylmalonate-semialdehyde dehydrogenase in the bands of mitochondrial (super)complexes after the PTP opening. Samples were obtained from the RHM and RLM incubated in SM-BM and RLM incubated in KCl-BM in the absence (Control samples) and presence of Ca^{2+} (PTP samples). Data from the corresponding raw MS datasets were extracted by PEAKS Studio 7.5/XPro. In pairs where a protein was absent (ND) either in a control or a PTP sample, the rIBAQ value is given for the present protein. H and L indexes at the CI-$CIII_2$-CIV_x supercomplex indicate high and low molecular weight of the latter.

	**rIBAQ PTP/rIBAQ Control**																	
	**RHM SM-BM**						**RLM SM-BM**						**RLM KCl-BM**					
	**CI-$CIII_2$-CIV_x (H)**	**CI-$CIII_2$-CIV_x (L)**	**CV_2**	**CI**	**$CIII_2$-CIV_x**	**CV**	**CI-$CIII_2$-CIV_x (H)**	**CI-$CIII_2$-CIV_x (L)**	**CV_2**	**CI**	**$CIII_2$-CIV_x**	**CV**	**CI-$CIII_2$-CIV_x (H)**	**CI-$CIII_2$-CIV_x (L)**	**CV_2**	**CI**	**$CIII_2$-CIV_x**	**CV**
**Prohibitin 2**	0.650	0.834	0.184	1.019	+ / (0.29)	ND	1.298	0.755	0.512	0.584	ND	ND	0.016	0.747	0.013	0.183	ND	ND
**Prohibitin**	0.334	0.158	0.507	0.464	+ / (0.085)	ND	0.936	0.571	0.609	1.063	2.712	ND	0.742	0.880	0.834	0.159	ND	ND
**Methylmalonate-semialdehyde dehydrogenase**	- / (0.36)	ND	- / (0.12)	ND	0.115	ND	0.013	0.012	0.028	0.11	0.244	0.401	0.010	0.018	0.029	0.165	0.112	0.348

Dataset S1. **Mass-spectrometry data of protein content of F-ATP synthase monomer and dimer bands from control samples of RHM. Raw data were processed using Thermo Xcalibur Qual Browser software.**

Dataset S2. Mass-spectrometry data of protein content of F-ATP synthase monomer and dimer bands from PTP samples of RHM. Raw data were processed using Thermo Xcalibur Qual Browser software.

Dataset S3. Mass-spectrometry data of protein content of F-ATP synthase monomer and dimer bands from Control samples of RLM incubated in KCl-BM. Raw data were processed using Thermo Xcalibur Qual Browser software.

Dataset S4. Mass-spectrometry data of protein content of F-ATP synthase monomer and dimer bands from PTP samples of RLM incubated in KCl-BM. Raw data were processed using Thermo Xcalibur Qual Browser software.

Dataset S5. Mass-spectrometry data of protein content of F-ATP synthase monomer and dimer bands from Control samples of RLM incubated in SM-BM. Raw data were processed using Thermo Xcalibur Qual Browser software.

Dataset S6. Mass-spectrometry data of protein content of F-ATP synthase monomer and dimer bands from PTP samples of RLM incubated in SM-BM. Raw data were processed using Thermo Xcalibur Qual Browser software.

Dataset S7. Comparison of protein composition of F-ATP synthase dimers and monomers from control and PTP samples. Samples were obtained from the RHM and RLM incubated in SM-BM and RLM incubated in KCl-BM in the absence (Control samples) and presence of Ca^{2+} (PTP samples). The table summarizes the data from Datasets S1-S6 extracted by PEAKS Studio 7.5/XPro. Sheet titles indicate F-ATP synthase form (Dimer or Monomer), mitochondrial type (RHM or RLM), sample type (Control or PTP), incubation medium (SM-BM or KCl-BM), and whether the list contains information about all true proteins (All) or only those whose PTP/control rIBAQ ratio is greater than two (Big Difference).

Dataset S8. Protein composition of high- (H) and low-molecular weight (L) CI-$CIII_2$-CIV_x supercomplexes and $CIII_2$-CIV_x supercomplex from control and PTP samples isolated from RHM. Data from the corresponding raw MS datasets were extracted by PEAKS Studio 7.5/XPro. Sheet titles indicate the supercomplex and sample type.

Dataset S9. Protein composition of high- (H) and low-molecular weight (L) CI-$CIII_2$-CIV_x supercomplexes and $CIII_2$-CIV_x supercomplex from control and PTP samples isolated from RLM incubated in SM-BM. Data from the corresponding raw MS datasets were extracted by PEAKS Studio 7.5/XPro. Sheet titles indicate the supercomplex and sample type.

Dataset S10. Protein composition of high- (H) and low-molecular weight (L) CI-$CIII_2$-CIV_x supercomplexes and $CIII_2$-CIV_x supercomplex from control and PTP samples isolated from RLM incubated in KCl-BM. Data from the corresponding raw MS datasets were extracted by PEAKS Studio 7.5/XPro. Sheet titles indicate the supercomplex and sample type.
