# Supplementary figures and images for: Mitochondrial F-ATP Synthase Co-Migrating Proteins and Ca2+-Dependent Formation of Large Channels
Source: Cells. 2023 Oct 7;12(19):2414. doi: 10.3390/cells12192414 (PMC10572550; doi:10.3390/cells12192414)

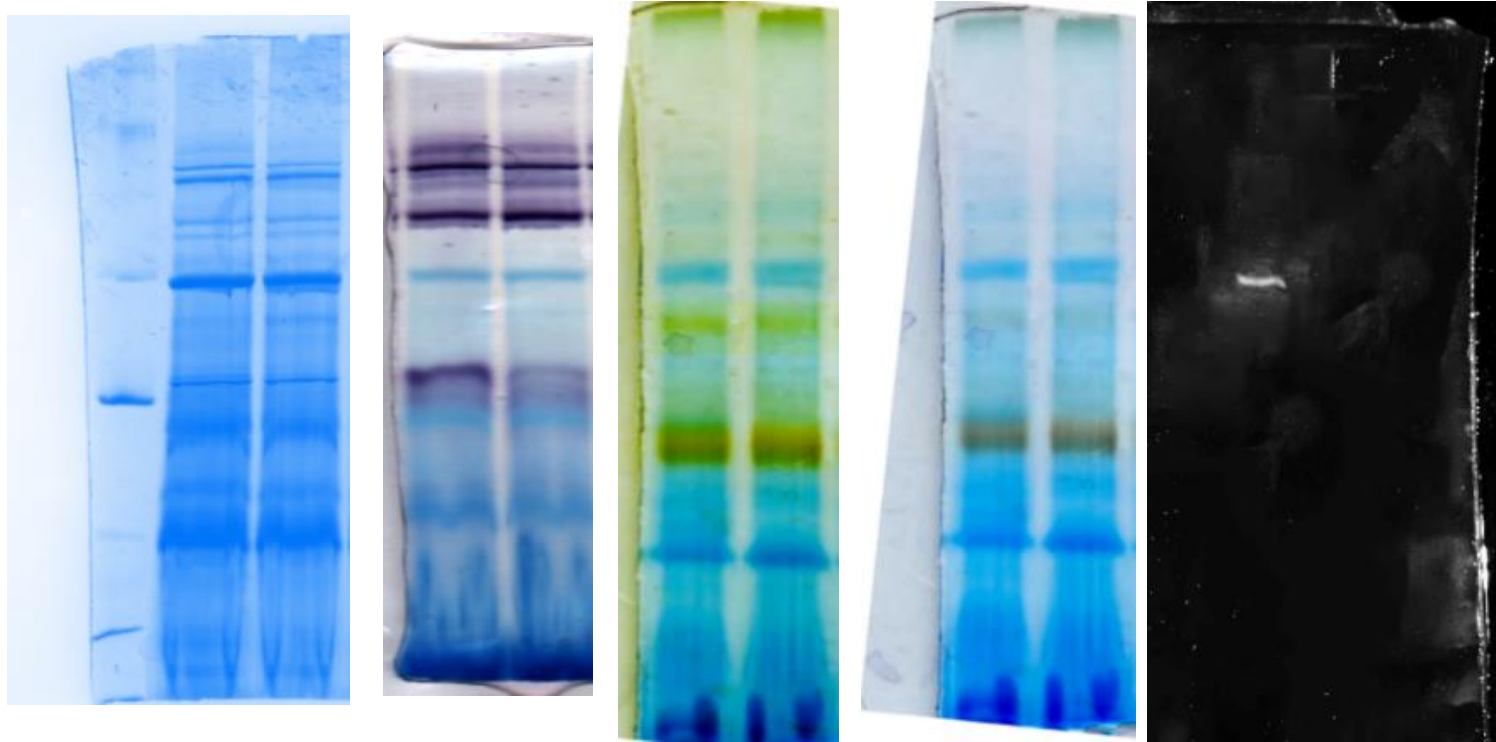

To Fig 1

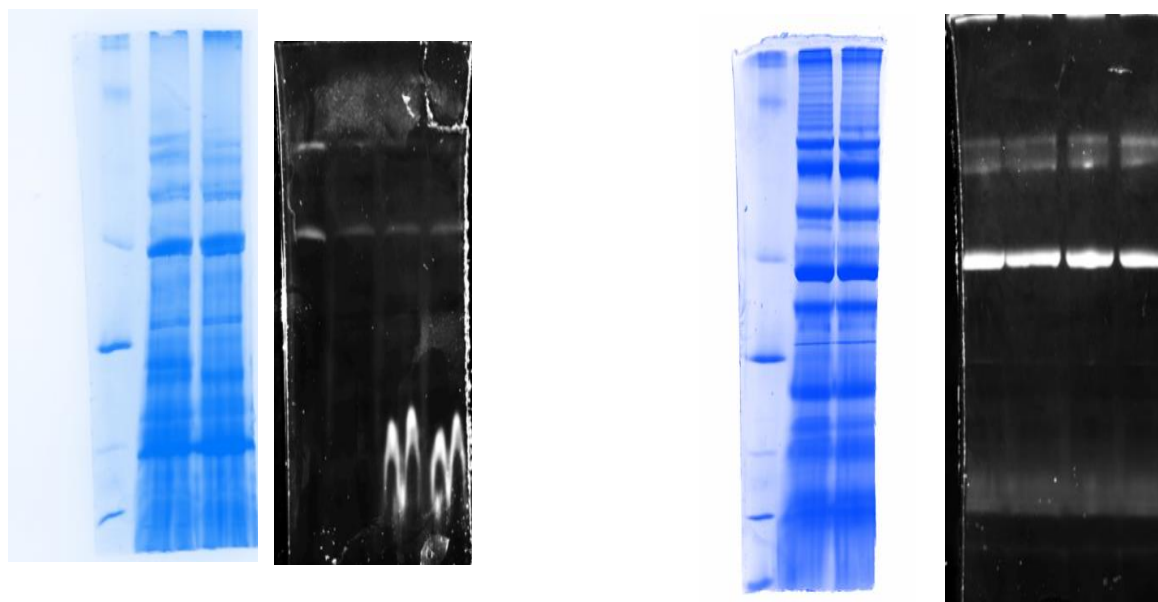

To Supl

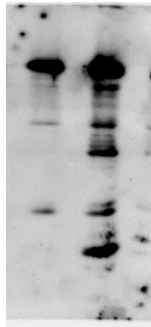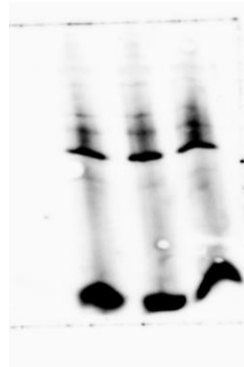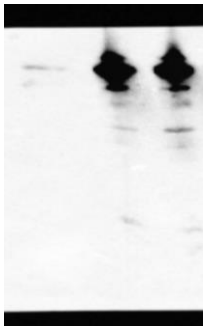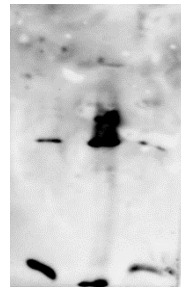

Supplement: Supplementary file 1 [file cells-12-02414-s001.zip › blots Kruglov.pdf]
